# Łucja Frey (1889–1942)

**DOI:** 10.1007/s00415-016-8107-2

**Published:** 2016-04-02

**Authors:** Andrzej Grzybowski, Jarosław Sak

**Affiliations:** 1 Department of Ophthalmology, Poznań City Hospital, ul. Szwajcarska 3, Poznań, 61-285 Poland; 2 Medical Faculty, University of Warmia and Mazury, Olsztyn, Poland; 3 Department of Ethics and Human Philosophy, Medical University of Lublin, ul. S. Staszica 4-6 (Collegium Maximum), Lublin, 20-059 Poland

**Fig. 1:**
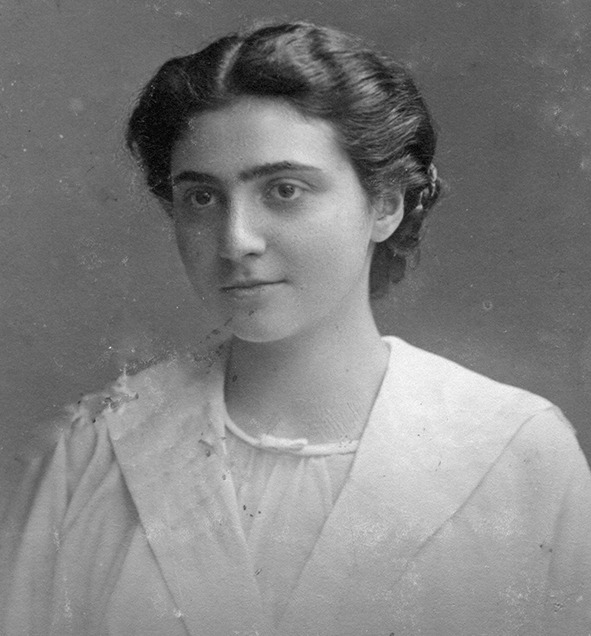
Łucja Frey in 1918. The photo is from the first page of “Index Lectionum” of Lviv University No. 4977 (from the Archives of the University of Warsaw)

Łucja Frey (Fig. 1) was a Jewish neurologist who described and explained the pathomechanism of the auriculotemporal nerve syndrome—“Frey’s syndrome” [1–3].

Łucja Frey was born on November 3, 1889 in Lviv (now western Ukraine). Lviv was an important multicultural and scientific center in pre-war Poland, i.e. in the times of Second Polish Republic (1918–1939). She was the daughter of Szymon Symcha Frey who was a building contractor and Dina née Weinreb [4, 5]. Łucja Frey’s parents were assimilated Jews.

In 1900, she finished Catholic elementary school in the Benedictine monastery. The next stage of her education included a 7-year period starting in 1900 when she attended the Kämmerling Goldblatt Jewish School for girls.

Łucja Frey started studying at the University of Lviv after passing the Matura exam. Initially, she studied philosophy in 1908 and later she changed the course of study to mathematics [4, 5]. Łucja Frey passed her license teaching exams in 1913. She started medical studies in Lviv 4 years later—in 1917, but due to the Polish-Ukrainian war she abandoned her medical education for one academic year between 1918/1919.

During the war Frey worked in the neuropsychiatric ward of the National Hospital in Lviv under the tutelage of Kazimierz Orzechowski (1878–1942). After finishing four semesters of medical studies in Lviv, Łucja Frey continued her studies in Warsaw. She worked as a junior assistant at the university Department of Neurological Diseases in Warsaw [4, 5]. From 1920, the management of the neurological clinic was undertaken by Kazimierz Orzechowski who was the founder of the Second Polish Neurological School [the founder of the so-called First Polish Neurological School was Edward Flatau (1868–1932) [5, 6]].

Łucja Frey received her medical diploma in 1923 at the age of 34. In the years between 1923 and 1928, she was a senior assistant in the neurological department in Warsaw [4–6]. In 1929, Łucja Frey returned to Lviv, where she worked in the Jewish Community Hospital, and married an attorney, Mordechaj Gottesman. Her daughter Danuta was born in 1930 and she probably also had a son, Jacob [4, 5].

After the outbreak of the Second World War in September 1939 and after the German and Soviet invasions of Poland, her husband was accused of anticommunist activity during the occupation of Lviv by Soviet armies. He was arrested by the Soviet secret police (N.K.V.D.) and probably murdered. In June 1941 after the outbreak of the German–Soviet war and the March of the German Army into Lviv, Łucja Frey together with her family and 136,000 other people of Jewish nationality were brought to live in the Ghetto Lemberg [5]. She started to work in the ghetto policlinic (the so called “Second Jewish Clinic”). In the period between March 1942 and June 1943 all Jewish habitants were transported from the ghetto to the Nazi death camps or were murdered in Lviv. On August 20, 1942, almost all the patients and medical staff of the Second Jewish Clinic were murdered. Łucja Frey died then or was deported between August 10 and 22 to the Nazi death camp in Bełżec (now South-East Poland) [4, 5, 7].

In spite of working in unusually difficult interwar and war times, Łucja Frey published many valuable original papers and case studies concerning neurological issues. One should emphasize the fact that she published the observation of the auriculotemporal nerve syndrome in the same year in which she finished medical studies, i.e. in 1923.

The case illustrating this syndrome which made the name of Łucja Frey famous in the medical literature was published in the Polish journal “Polska Gazeta Lekarska” [8] and then in the same year in the French journal “Revue Neurologique” [9]. She presented the case of 25 year-old man who was wounded on the left side of the lower mandible by a rifle bullet [5, 8, 9]. After regaining consciousness he noticed that the left side of his face was very swollen. A fistula in the internal auditory meatus, but without further damage to the tympanic membrane, was diagnosed by an otorhinolaryngologist as the probable cause of these manifestations about 3 months later. These symptoms entirely yielded after an incision in the place of the first wound. The patient then started complaining about the following manifestations: redness of the left side of his face, and a feeling of warmth and sweating in this area while he was eating. The patient was ashamed to eat meals in public places stating that “people think that I ate so voraciously” [5, 8, 9]. Łucja Frey carried out pharmacological provocation tests, and became convinced that the injection of 1 mg of atropine caused a significant dryness of the mouth, a lack of redness of the skin of the lower mandible, and it eliminated the sweating. Paleness and a reduction of warmth of the left side of the face was caused by hypodermic injection of 1 mg of Physostigmine [5].

Łucja Frey was not the first to present a description of this syndrome in the medical literature [10] but she gave a systematic pathophysiological explanation with reference to all the observed symptoms as a disorder of both parasympathetic and sympathetic nerves and determined by anatomical relations of the sensory and the autonomic innervation of the head [5, 8, 9].

Apart from papers on the auriculotemporal nerve syndrome, Łucja Frey published works in the Polish medical journals “Neurologia Polska” and “Gazeta Lekarska” (in the years 1925–1928), which concerned brain stem topography, amyotrophic lateral sclerosis (Charcot’s disease), aneurysms of the plexus of the medulla, clivus tumors, frontal lobe tumors and retrosplenial tumors [5].
